# Exploring the intersectionality of family SES and gender with psychosocial, behavioural and environmental correlates of physical activity in Dutch adolescents: a cross-sectional study

**DOI:** 10.1186/s12889-022-13910-6

**Published:** 2022-08-27

**Authors:** André Mamede, Özcan Erdem, Gera Noordzij, Inge Merkelbach, Paul Kocken, Semiha Denktaş

**Affiliations:** 1 Department of Psychology, Education and Child Development, Erasmus School of Social and Behavioural Sciences, Erasmus University Rotterdam, Rotterdam, The Netherlands; 2 Municipality of Rotterdam, Department Research and Business Intelligence, Rotterdam, The Netherlands; 3 Erasmus University College, Erasmus School of Social and Behavioural Sciences, Erasmus University Rotterdam, Rotterdam, The Netherlands

**Keywords:** Exercise, Youth, Health behaviour, Risk factors, Multilevel analysis, Social support

## Abstract

**Background:**

Examining the correlates of adolescent’s physical activity (PA) and how they may differ according to the intersection of gender and family socioeconomic status (SES) can support the development of tailored interventions to more effectively promote adolescents’ PA. This study explored how the associations between psychosocial, behavioural and environmental factors and adolescent’s PA differed according to gender and family SES.

**Methods:**

This study used data from the Dutch Youth Health Survey 2015. Adolescents (*n* = 9068) aged 12–19 were included in the study. The associations between psychosocial, behavioural, and environmental factors and PA (days per week engaging in at least one hour of PA) were examined with multilevel linear regression analysis. Potential interactions between these correlates, gender and family SES were explored.

**Results:**

On average, adolescents engaged in at least one hour of PA for 4,2 days per week. Poor self-perceived health, low peer social support, and a weak connection with the environment were all associated with lower PA in adolescents. Daily smoking, cannabis use, risk of problematic gaming and social media use, as well as lack of daily consumption of fruit, vegetables, water and breakfast were associated with lower PA, whereas binge drinking was not. Interactions revealed that poor self-perceived health was associated with lower PA in adolescents from moderate- and high-SES families, but not in low-SES adolescents, whereas cannabis use was only associated with lower PA amongst low-SES adolescents. Low peer social support was associated with lower PA across all groups, but it was most strongly associated with lower PA amongst male adolescents from low-SES families than in other subgroups. Amongst low-SES males, low peer social support was associated with a 1.47 reduction in days engaging in sufficient PA, compared with a 0.69 reduction for high-SES males.

**Conclusions:**

This study identified several psychosocial, behavioural and environmental factors that can be targeted to potentially increase adolescent’s PA. We also found that correlates of PA differed according to the intersection of gender and family SES. Our findings suggest that PA interventions should be tailored according to gender and SES to address the specific needs, barriers and facilitators of different subgroups.

**Supplementary Information:**

The online version contains supplementary material available at 10.1186/s12889-022-13910-6.

## Introduction

Regular engagement in moderate-to-vigorous physical activity (MVPA) is associated with increased quality of life [1] and contributes to the prevention of obesity, cardiovascular disease, cancer and depression [2, 3, 4]. Given adolescent physical activity (PA) tracks into young adulthood and influences life-long patterns of healthy behaviours [5], it is essential to ensure that adolescents engage in sufficient PA. However, less than half of adolescents adhere to MVPA guidelines (i.e. one hour per day) in most western countries, such as in the USA (32.6% of boys and 20.1% of girls) and in the Netherlands (22.3% of boys and 15.7% of girls). Evidence suggests that adolescents in families of low-socioeconomic status (SES) are even less likely to adhere to MVPA guidelines [6]. Adolescents’ PA seems to be influenced by complex interactions of psychosocial, behavioural, and environmental factors, and studies have shown that the link between these factors and PA may differ according to the adolescents’ SES and sociodemographic characteristics (e.g. gender) [7]. These findings highlight the need of not only identifying modifiable factors for insufficient PA in adolescents, but also to investigate how these factors may differ according to socio-demographic and -economic characteristics.

Socio-ecological models of behaviour change suggest that health behaviours, such as PA, are influenced by the interaction of factors at different levels [8, 9]. At the individual level, various socio-demographic and -economic characteristics, as well as behavioural and psychosocial factors have been identified as important correlates of PA. Research has shown that immigrant background, lower SES, female gender and older age in adolescents, were all predictors of lower levels of PA [6, 10, 11]. Regarding behavioural factors, adolescent PA has been shown to be negatively associated with several health behaviours, such as lower fruit and vegetable consumption and cigarette smoking [12], as well as with excessive screen-time behaviours, such as problematic gaming [13] and social media use [14]. Adolescent PA has also been negatively associated with psychosocial factors, such as poor self-perceived health status [15] and lower levels of social support from peers and family [16, 17]. Besides individual-level correlates, several environmental factors have been linked with adolescents’ PA, including objective factors such as availability of recreational facilities [18], but also self-perceived factors such as aesthetics [18] and social ties with the neighbourhood [19]. Remarkably, socio-ecological models propose not only that factors in multiple levels influence behaviour, but also that these factors interact. For example, the link between environmental factors (e.g. safety) and PA may differ according to someone’s gender or to how much social support for PA they receive from their peers [8].

Several approaches have emerged in different fields emphasizing the need to explore interactions between factors at different levels to better explain health inequalities. One sociological approach that has recently been applied to epidemiological studies is intersectionality theory [20]. Intersectionality theory posits that a person is shaped by their experiences as a whole. Thus, by dividing and addressing single categories of identity, key interactions between multiple characteristics of an individual’s identity that create unique experiences may be overlooked. Therefore, it may be essential to consider how correlates of PA may differ according to these unique experiences that are largely shaped by the interplay of socio-demographic and -economic characteristics (e.g. gender, SES, ethnicity). For example, one study found that the positive effect of household income on PA was strong among ethnic minority men, but almost non-existent among ethnic minority woman [21]. Similarly, the relative importance of certain psychosocial, behavioural and environmental factors of PA may differ significantly between subgroups. Given that correlates of adolescent’s PA seem to vary according to gender [7, 22] and SES [23], it is plausible that the link between certain factors and PA may depend on the unique interaction of adolescents’ socio-demographic and -economic characteristics.

Although some studies have explored whether certain correlates of PA in youth differed according to gender or SES, these studies have typically focused on only a few correlates [24] or examined differences between genders or SES levels separately [25, 26], rather than exploring how various correlates of PA may vary according to the interplay of gender and SES in a large sample of adolescents. Examining how correlates of adolescent’s PA may differ according to their socio-demographic and -economic subgroup could facilitate the development of tailored PA interventions that address the unique needs of the individual. Therefore, in this explorative study we analysed data from a survey to identify factors associated with adolescent’s PA and possible interactions with gender and family SES. We aimed to explore whether behavioural (e.g. smoking), psychosocial (i.e. self-perceived health and peer social support), and environmental factors (i.e. connection with neighbourhood) correlate independently with adolescent’s PA. Additionally, this study explores whether the association between these factors and PA differ according to the intersection of gender and family SES, which have been identified as the most relevant socio-demographic and -economic factors for PA [22, 27, 28]. We expect to find associations between PA in adolescents and the factors selected based on the literature [12, 13, 19, 29]. Additionally, we hypothesize that the correlates of PA in adolescents will vary according to the interplay of gender and family SES, seeing as the intersection of these socio-demographic and -economic characteristics constitutes significantly different groups and identities.

## Methods

### Survey design and population

The present study concerns a secondary analysis utilizing the data from the Youth Health Survey 2015 *(Gezondheidsmonitor Jeugd 2015 GGD’en en RIVM)* conducted by the Municipal Health Services of Rotterdam in the Netherlands [30]. This survey was conducted to assess the mental and physical health of adolescents (aged 12–19 years), and to gain insight into the correlates of health behaviours in this population. This survey was administered in Rotterdam, the second largest city of The Netherlands, and in 14 smaller surrounding municipalities. A total of 50 secondary schools participated in this survey, corresponding to approximately 39% of the secondary school population in Rotterdam and surrounding municipalities. Data was collected in the classroom on a voluntary basis among students in grades 2 and 4 (equivalent to grades 8 and 10 in the United States) in the fall of 2015. Parents received written information on the survey and could object to their child’s participation. In total 9136 adolescents participated in the study. The response rate was 76%. For the analyses we included adolescents (*n* = 9068) who filled out the key variables (gender, age, migration background, educational level, family SES, municipality and physical activity). According Dutch law, research that does not subject participants to procedures or require them to behave in a particular way does not require approval of an ethics committee [31]. This study relied on secondary analysis of anonymized data and was therefore exempt from ethical approval. The Dutch Code of Conduct for Medical Research allows the use of anonymous data for research purposes without an explicit informed consent from the participants.

### Measures

The survey combined validated questionnaires and scales jointly developed for the Youth Health Survey by all municipal health services in the Netherlands in collaboration with the National Institute for Public Health and the Environment. Unless otherwise specified, the scales and cut-off points used were jointly designed and tested. The questionnaires developed for this survey have been continuously tested and improved, and these scales have now been widely used in other research based on data from the Youth Health Survey [32, 33]. All the questionnaires were administered in Dutch.

### Outcome measure

*Physical activity* included both moderate and vigorous physical activity (MVPA), and was assessed with the item “How many days per week do you do sports or physical exercise for at least 1 hour?”. This item was scored on an 8-point Likert scale, ranging from “(0) (Almost) never” to “ [7] Everyday”. Adolescents were instructed to add physical activity for all purposes throughout the day, and examples were provided for moderate and vigorous physical activities (e.g. biking to school, sports). This item was adapted to the Dutch context and was similar to the item used in the WHO Collaborative Health Behaviour in School-aged Children (HBSC) studies [34], as well as to the two-item questionnaire developed by Prochaska and colleagues [35], which also asked for physically active days in the past week. The original version of Prochaska’s questionnaire had acceptable test-retest reliability (ICC = 0.77) and validity, as questionnaire responses were moderately correlated with PA objectively measured through accelerometers (*r* = 0.40). Validation studies of PA measures similar to the one used in the present study have shown that such measures have acceptable test-retest reliability and validity for assessing adolescent’s MVPA and achievement of physical activity guidelines [36, 37].

### Socio-demographic measures

*Demographics characteristics* measured included gender (m/f), age (year of birth), migration background and educational level. Gender was determined by asking adolescents whether they were a boy or a girl. Migration background was determined by asking the birthplace of adolescents and their parents, and participants were categorized as either native Dutch (i.e. both parents and the adolescent were born in the Netherlands), Western immigrants (i.e. the adolescent, or one or both parents were born in a Western country) or non-Western immigrants (i.e. the adolescent, or one or both parents were born in a non-Western country). Educational level was measured by asking participants to indicate their current educational level (i.e. basic or theoretical pre-vocational secondary education, secondary education, pre-university education).

### Family SES

*Perceived financial difficulties* were used as an indicator of family SES and were measured with the following item developed “Do you have difficulties to make ends meet at home with regards to money? (by home we mean the family that you live with most of the time)” rated on a 4-point Likert scale. Answering options included “No, we do not have difficulties at all”, “No, we do not have difficulties but we must be careful with our spending”, “Yes, we have some difficulties” and “Yes, we have a lot of difficulties”. Family SES was categorized as High if they answered “No, we do not have difficulties at all”, Moderate if they answered “No, we do not have difficulties but we must be careful with our spending”, and Low if they answered either “Yes, we have some difficulties” or “Yes, we have a lot of difficulties”.

### Behavioural factors

*Food and Water intake* was assessed through multiple variables related to eating and drinking behaviour. To measure daily fruit and breakfast consumption, single-item questions from previous research assessing these eating behaviours in adolescents in the Netherlands were used [37]. *Daily fruit consumption* was assessed with the item “On how many days per week do you eat fruit?” and *daily breakfast consumption* was assessed with the item “On how many days per week do you eat breakfast?”, both which were rated on an 8-point Likert scale indicating the number of days ranging from “(Almost) never” to “Every day”. Based on these items, two additional single-item questions were developed for the Health Monitor Survey to measure daily vegetable consumption and daily water drinking. *Daily vegetable consumption* was assessed with the item “On how many days per week do you eat vegetables?” and *daily water drinking* was assessed with the item “On how many days per week do you drink one or more glasses of water?”, which were similarly rated on an 8-point Likert ranging from “(0) (Almost) never” to “ [7] Every day”. Daily food and water intake variables were dichotomized as Yes (i.e. participants who answered Every day) and No (i.e. participants who reported consumption on six days or less).

*Excessive screen time behaviours* were assessed by measuring *problematic social media use* and *problematic gaming*. The items used were adapted from the validated Compulsive Internet Use Scale [38] and the Gaming Addiction Scale [39]. *Problematic social media use* was assessed with six items, such as “How often do you find it difficult to stop using social media?” or “How often do you feel restless, stressed, or irritated when you cannot use social media?”. *Problematic gaming* was similarly assessed with six items, such as “How often do you find it difficult to stop gaming?” or “How often do you prefer to game than to spend time with others (e.g. with your friends or your parents)?”. Items were scored on a 5-point Likert scale ranging from “ [1] Never” to “ [5] Very often”. For both variables, participants’ responses to the six items were averaged, and participants whose average score was higher than two were categorized as having a risk for problematic gaming. Participants were also categorized as not having a significant risk for problematic gaming or social media use if they answered “(Almost) Never “ to the precursor items: “How often do you game?” or “How often are you active on social media?”. *Problematic gaming* and *social media use* scales were developed by IVO [40], an institute responsible for researching problematic internet use and health behaviours in youth in the Netherlands.

*Early use of drugs and alcohol* was assessed through several items measuring the frequency of consumption of common soft drugs. *Daily smoking* was assessed by asking adolescents who indicated having smoked before to answer the item “How often do you smoke now” rated on a 4-point Likert scale with the following answering options: “I do not smoke”, “Less than one time per week”, “At least once per week, but not every day” “Every day”. Since we were interested in identifying clinically significant unhealthy behaviours as risk factors, daily smoking consumption was dichotomized as Yes (i.e. participants who reported smoking every day) and No (i.e. non-smokers and nondaily smokers) in order to distinguish between occasional smokers and heavy daily smokers. *Binge drinking* was assessed with the item “How often in the last 4 weeks did you consume five or more alcoholic drinks on one occasion (for example at a party on one evening?)”. The item was rated on 7-point Likert scale ranging from “Never” to “9 times or more”, and participants were categorized as binge drinkers if they answered any option other than “Never”, which would indicate at least one instance of binge drinking behaviour in the last four weeks. *Cannabis use* was assessed with the item “On how many days in the last four weeks did you consume weed (marijuana) or hash?”, which was rated on a 7-point Likert scale ranging from “Never” to “30 days or more”. Adolescents who indicated using cannabis in the previous four weeks were considered as cannabis users.

### Psychosocial factors

*Self-perceived health* was assessed with the validated single-question item “How is your health in general?” [41], which was rated on a 5-point Likert scale ranging from “ [1] Very bad” to “ [5] Very good”. This measure has been demonstrated to have predictive validity for several health outcomes, including all-cause mortality [42]. This variable was dichotomized into poor (i.e. “fair”, “bad”, and “very bad”) and good (i.e. “very good” and “good”) health, with good serving as the reference category.

*Peer social support* was assessed with six items from the validated European KIDSCREEN-52 health-related quality of life (HRQoL) questionnaire [43, 44] inquiring about their social experiences in the previous week. Examples of items used included: “Have you spent time with your friends?” or “Do you have friends who you can trust?”. Items were rated on a 5-point Likert scale indicating the frequency the behaviour/feelings in the statements (“Never”, “Seldom”, “Sometimes”, “Often” and “Always”). Based on the procedure of studies assessing the psychometric properties of KIDSCREEN-52, Rasch scores were computed for the *Social Support and Peers* variable and transformed into T-values, with a scale mean of 50 and a standard deviation of 10, with higher values indicating greater levels of peer social support. Subsequently, following the recommended thresholds for scoring KIDSCREEN-52 scales [45], the *Peer Social Support* variable was dichotomized as low when T-values were more than half a standard deviation below the mean (i.e. T-values < 45) or moderate-to-strong when T-values were not lower than half a standard deviation below the mean (i.e. T-values ≥45).

### Environmental factors

*Connection with neighbourhood* was assessed through three items, namely “I would like to move from my neighbourhood”, “If I were to move, I would really miss my neighbourhood” and “I think that I live in a great neighbourhood”, anchored on a 5-point Likert scale ranging from “ [1] Strongly agree” to “ [5] Strongly disagree”. After reversing the scores of the first item, a total score was calculated by averaging summing these three items, and this variable was subsequently dichotomized into weak connection with neighbourhood (i.e. a total score higher than 3.5) or strong-to-moderate connection with neighbourhood (i.e. a total score lower than 3.5).

### Statistical analysis

We analysed the descriptive characteristics of our study population stratified by gender (Table 1). In this study, observations of adolescents’ levels of PA (level 1) were nested within different schools (level 2), which in turn were nested in different municipalities (level 3). For that reason, we utilized multilevel linear models to account for the nested hierarchical structure of our data by including a random intercept in our model for the different levels when the variance at that level was significantly different from zero. Given that variance at the municipality level was not significant (*p > 0.05*), whereas variance at the school-level was significant (*p < 0.05*), a random intercept was included to adjust for the clustering effects of observations within the same school. The intraclass correlation coefficient (ICC) in the null model showed that 3.6% of the variation within adolescents’ PA is attributable to differences between the schools.

**Table 1:** Descriptive statistics of the study sample stratified by gender (*n* = 9068)

	Total		Males	Females
	Mean (S.D.)	Min-Max	Mean (S.D.)	Mean (S.D.)
**Outcome variable**				
*Physical activity*				
Number of days per week engaging in at least one hour of physical activity	4,2 (2,1)	(0–7)	4,4 (2,0)	3,9 (2,1)
**Family SES**				
Low	730	8.1	7.1	9.0
Middle	2861	31.6	28.9	34.3
High	5477	60.4	64.0	56.7
**Socio-demographic characteristics**				
*Gender*				
Males	4577	50.5	100.0	
Females	4491	49.5		100.0
*Age*				
≤ 13 years	3321	36.6	34.8	38.5
14 years	1591	17.5	18.6	16.5
15 years	2420	26.7	25.4	28.0
≥ 16 years	1736	19.1	21.3	17.0
*Migration background*				
Non-Western immigrants	3030	33.4	33.4	33.4
Western immigrants	677	7.5	7.8	7.1
Native Dutch	5361	59.1	58.8	59.5
*Educational level*				
Basic pre-vocational secondary education	3017	33.1	34.2	32.3
Theoretical pre-vocational secondary education	2757	30.4	29.9	30.9
Secondary education	1541	17.0	17.0	17.0
Pre-university education	1753	19.3	18.9	19.8
*Municipality*				
Rotterdam	3339	36,8	36,3	37,4
Surrounding municipalities	5729	63,2	63,7	62,6
**Behavioural factors**				
*Risk of problematic social media use*				
No or very little risk	7761	87.0	92.0	82.0
High risk	1156	13.0	8.0	18.0
*Risk of problematic gaming*				
No or very little risk	8299	93.3	88.4	98.2
High risk	597	6.7	11.6	1.8
*Daily smoking*				
No	8665	95.6	96.1	95.1
Yes	398	4.4	3.9	4.9
*Cannabis use in the last 4 weeks*				
No	8640	95.4	94.0	96.8
Yes	417	4.6	6.0	3.2
*Binge drinker*				
No	7652	84.4	84.3	84.6
Yes	1409	15.6	15.7	15.4
*Daily breakfast consumption*				
Yes	6161	68.0	72.4	63.4
No	2903	32.0	27.6	36.6
*Daily fruit consumption*				
Yes	2783	30.7	28.9	32.5
No	6279	69.3	71.1	67.5
*Daily vegetable consumption*				
Yes	3672	40.5	37.6	43.5
No	5393	59.5	62.4	56.5
*Daily water drinking*				
Yes	5678	62.6	62.1	63.1
No	3390	37.4	37.9	36.9
**Psychosocial factors**				
*Peer social support*				
High score on social support	8289	92.0	90.3	93.6
Low score on social support	725	8.0	9.7	6.4
*Self-perceived health*				
Good or very good	8040	88.7	91.1	86.3
Bad or very bad	1024	11.3	8.9	13.7
**Environmental factors**				
*Connection with the neighbourhood*				
Strong connection with the neighbourhood	7878	88.6	89.2	87.9
Weak connection with the neighbourhood	1018	11.4	10.8	12.1

We firstly investigated the relationship between different socio-demographic characteristics (i.e. gender, age, migration background, educational level, municipality), family SES and PA. Subsequently, we separately examined the relationships between psychosocial, behavioural and environmental factors and PA, while controlling for the effects of socio-demographics characteristics (i.e. gender, age, migration background, educational level, municipality) and family SES. Additionally, given that literature suggests that correlates of PA may differ by gender [7, 22] and SES [23, 46], we tested for possible two-way interactions between the psychosocial, behavioural and environmental factors and family SES and gender. If the two-way interaction term was statistically significant, the findings are described in the results and through a supplementary figure illustrating the interaction. Subsequently, we also tested for possible three-way interactions between the psychosocial, behavioural and environmental factors, family SES and gender. If three-way interaction term was statistically significant, we presented the associations in a table. All analyses were performed in SPSS 25. Results were considered to be statistically significant at *p* < 0.05.

## Results

### Study sample

Table 1 shows the descriptive statistics of our study sample stratified by gender.

### The associations of family SES and socio-demographic characteristics with PA in adolescents

Multilevel linear regression analysis showed that female gender, older age, low levels of education and low family SES were associated with decreased PA (Table 2). Additionally, native Dutch adolescents moved significantly more than both adolescents with Western immigrant and non-Western immigrant backgrounds, with non-Western immigrant background being associated with the lowest levels of PA.

**Table 2:** The associations between socio-demographic characteristics, family SES and levels of PA

	B^a^	(95% CI)	
**Intercept**	**5.25**	**5.05**	**5.46**
**Family SES**			
Low	**−0.32**	−0.47	−0.16
Medium	**−0.14**	−0.23	−0.05
High	*ref.*		
**Gender**			
Females	**−0.50**	−0.58	−0.41
Males	*ref.*		
**Age**			
≤ 13 years	**0.37**	0.25	0.49
14 years	**0.33**	0.19	0.47
15 years	**0.18**	0.06	0.31
≥ 16 years	*ref.*		
**Migration background**			
Non-Western immigrants	**−0.39**	− 0.49	− 0.29
Western immigrants	**− 0.25**	− 0.41	− 0.08
Native Dutch	*ref.*		
**Educational level**			
Basic pre-vocational secondary education	**−0.64**	− 0.81	− 0.48
Theoretical pre-vocational secondary education	**− 0.44**	− 0.59	− 0.28
Secondary education	**− 0.16**	− 0.31	0.01
Pre-university education	*ref.*		
**Municipality**			
Rotterdam	−0.06	− 0.17	0.05
Other municipalities	*ref.*		

^a^ Bold beta indicates statistical difference in mean PA relative to reference category (*p *< 0.05)

### Behavioural, psychosocial and environmental correlates of PA in adolescents

Regarding behavioural factors, multilevel linear regression analysis demonstrated that problematic social media use, problematic gaming, daily smoking, and cannabis use in the previous four weeks were all significant predictors of lower levels of PA in adolescents (Table 3). Conversely, our findings showed that daily water drinking, as well as daily breakfast, fruit and vegetable consumption, were all significant predictors of higher levels of PA. Additionally, with respect to psychosocial factors, adolescents with poor self-perceived health and low peer social support reported lower levels of PA. With regards to environmental factors, a weak connection with the neighbourhood was a predictor of lower PA.

**Table 3:** The associations between psychosocial, behavioural and environmental factors and PA in adolescents^b^

		B^a^	95% CI	
**Behavioural factors**				
** Model 1**	Lack of daily fruit consumption (ref. = daily consumption)	**− 0.48**	− 0.57	− 0.39
** Model 2**	Lack of daily vegetable consumption (ref. = daily consumption)	**− 0.33**	− 0.43	− 0.25
** Model 3**	Lack of daily water drinking (ref. = daily consumption)	**− 0.44**	−0.53	− 0.35
** Model 4**	Lack of daily breakfast consumption (ref. = daily consumption)	**−0.33**	−0.42	− 0.24
** Model 5**	Risk of problematic social media use (ref. = no risk)	**−0.34**	−0.46	− 0.21
** Model 6**	Risk of problematic gaming (ref. = no risk)	**−0.53**	−0.69	− 0.35
** Model 7**	Daily smoking (ref. = not smoker)	**−0.31**	−0.52	− 0.11
** Model 8**	Cannabis use in the last 4 weeks (ref. = no use)	**−0.21**	− 0.41	−0.01
** Model 9**	Binge drinking (ref. = no binge drinking)	0.03	−0.09	0.15
**Psychosocial factors**				
** Model 10**	Poor self-perceived health (ref. = good)	**−0.50**	− 0.63	−0.37
** Model 11**	Low peer social support (ref. = high)	**−0.71**	−0.86	− 0.56
**Environmental factors**				
** Model 12**	Weak connection with the neighbourhood (ref. = strong)	**−0.21**	−0.35	− 0.08

^a^ Bold beta indicates statistical difference in mean PA relative to reference category (*p *< 0.05)

^b^ Each model was adjusted for gender, age, migration background, educational level, municipality and family SES

### Exploring the interactions between family SES, gender and correlates of PA

Our results indicate that the association between certain factors and adolescent’s PA varies according to gender and/or family SES, as evidenced by two-way and three-way interactions detected in our models.

#### Cannabis

A two-way interaction revealed that the association between cannabis use and PA was significantly different for adolescents with different levels of family SES. Namely, as shown in Supplementary Fig. S1, cannabis use in the last four weeks was only significantly associated with lower PA for adolescents from low-SES families (*B* = − 0.72, 95% CI [− 1.30, − 0.14]), but not for those from moderate (*B* = − 0.31, 95% CI [− 0.68, 0.06]) and high (*B* = − 0.03, 95% CI [− 0.29, 0.24]) SES families. No significant three-way interaction between gender, family SES and cannabis use was observed.

#### Perceived health

A two-way interaction revealed that the association between self-perceived health and PA was significantly different for adolescents with different levels of family SES. As shown in Supplementary Fig. S2, poor self-perceived health was only negatively associated with PA in adolescents from high- (*p* < 0.05, *B* = − 0.57, 95% CI [− 0.76, − 0.37]) and moderate- (*p* < 0.05, *B* = − 0.62, 95% CI [− 0.83, − 0.40]) SES families, but not in adolescents from low-SES families (*p* > 0.05, *B* = − 0.12, 95% CI [− 0.48, 0.23]). A two-way interaction also indicated that the association between self-perceived health and PA was also significantly different between genders. As shown in Supplementary Fig. S3, poor self-perceived health was a significantly stronger risk factor for lower PA in males (*p* < 0.05, *B* = − 0.68, 95% CI [− 0.88, − 0.48]) than in females (*p* < 0.05, *B* = − 0.33, 95% CI [− 0.51, − 0.16]). No significant three-way interaction between gender, family SES and self-perceived health was observed.

#### Peer social support

A two-way interaction (not shown) suggested that low peer social support seemed to have a stronger association with PA for adolescents from low- or moderate-SES families than for adolescents from high-SES families, although this interaction did not reach statistical significance (*p =* 0.11, *t* = 1.59, 95% CI [− 0.04, 0.38]). The analysis revealed a borderline significant three-way interaction between peer social support, family SES and gender (*p* = 0.052, *t* = 1.94, 95% CI [− 0.01, 0.20]), indicating that the effect of peer social support on PA differed according to the interplay of gender and SES. Table 4 shows the associations between peer social support and PA in adolescents stratified by family SES and gender. The model indicated that low peer social support had a stronger negative association with PA amongst male adolescents from low-SES families than in other subgroups. Amongst low-SES males, low peer social support was associated with a 1.47 reduction in days engaging in sufficient PA, compared with, for example, a 0.69 reduction for males from high-SES families, and with a 0.8 days reduction for females from low-SES families (See Table 4).

**Table 4:** The associations between peer social support and levels of PA in adolescents stratified by family SES and gender^b^

Family SES	Gender	Peer Social Support	B^a^	(95% CI)	
Low	Males	Low peer social support (ref. = high)	**-1.47**	-2.05	-0.90
	Females	Low peer social support (ref. = high)	**-0.80**	-1.47	-0.14
Moderate	Males	Low peer social support (ref. = high)	**-0.85**	-1.19	-0.52
	Females	Low peer social support (ref. = high)	**-0.42**	-0.81	-0.03
High	Males	Low peer social support (ref. = high)	**-0.69**	-0.95	-0.44
	Females	Low peer social support (ref. = high)	**-0.52**	-0.88	-0.15

^a^ Bold beta indicates statistical difference in mean PA relative to reference category (*p *< 0.05)

^b^ Model adjusted for age, migration background, educational level and municipality

## Discussion

This study explored socio-demographic, socio-economic, behavioural, psychosocial, and environmental correlates of adolescent PA and examined whether the correlates of PA varied according to the intersection of gender and family SES.

Our findings for socio-demographic and -economic factors revealed that female gender, older age, lower family SES, lower educational levels and immigrant backgrounds were all associated with lower PA. These results support previous findings linking lower PA in adolescents to female gender, older age, immigrant backgrounds, lower family SES and lower education [6, 10, 27, 46]. Several mechanisms could explain these associations. For example, research shows that self-efficacy and social support for PA are key mediators of the relationships between PA and both income and educational level [46].

Amongst behavioural factors, daily smoking, cannabis use, risk of problematic gaming and social media use, as well as lack of daily consumption of fruit, vegetables, water and breakfast were all associated with lower PA. These findings are in line with the evidence that adolescent PA are associated with other health behaviours, such as smoking [12], and that motivational and self-regulatory processes leading to engagement in one’s health behaviour may “spill-over” into another [47, 48]. Such psychosocial factors are also likely confounders, given that higher self-efficacy, social support or self-regulatory capacity are all related to increased PA [49, 50, 51], but also predict, for example, greater vegetable consumptions [52, 53] or less smoking [54, 55]. Our findings support the evidence linking problematic social media use or gaming to decreased PA [13, 14]. Although confounding variables could partially explain this association, problematic social media use and gaming may have a more direct negative effect on PA, since they can increase sedentary behaviour and substitute PA in the adolescent’s routine [56, 57].

For psychosocial factors, poorer self-perceived health and lower peer social support were associated with lower PA. Our findings that poorer self-perceived health is associated with lower PA in adolescents are consistent with evidence from a recent systematic review [58]. This study also found that peer social support was strongly associated to PA, which is consistent with research showing that peer social support is one of the most important predictors of adolescent’s PA [50].

Finally, for environmental factors a weaker connection with the neighbourhood was associated with lower PA, which is consistent with studies demonstrating a relationship between a neighbourhood’s social environment and adolescent PA [19, 59]. The strong social ties in one’s neighbourhood may influence adolescent’s PA through several stress-buffering, health-enhancing mechanisms, such as social support, social norms and improved self-efficacy [60].

Although several epidemiological studies have explored correlates of PA in adolescents, few studies have explored how these correlates vary according to the interplay of certain socio-demographic and -economic characteristics. Intersectionality theory emphasizes that people’s resources, experiences and identities are not only shaped by several individual characteristics, such as gender and SES, but also by the unique interplay of these characteristics [61]. From the lens of intersectionality, low SES and female gender are associated with vulnerabilities for lower PA which may not be separate, but rather interactive and multiplicative in their effects [62]. Our findings indicated that the association between certain factors and PA varied according to the intersection of gender and family SES, which may reflect the unique needs and barriers for PA in subgroups with compounding vulnerabilities.

While most behaviours were consistently related to PA across gender or family SES in our sample, our findings showed that cannabis use was only associated with lower PA amongst adolescents from low-SES. This may reflect the role of possible confounders, such as low self-control or low parental social support, which may render adolescents with low-SES more vulnerable to both early cannabis use and low levels of PA [17, 55, 63, 64]. Future research is needed to examine the causality of the relationship between early cannabis use and adolescent PA, while accounting for potential confounders.

Furthermore, in our sample, poorer perceived health was associated with lower PA in adolescents from moderate- or high-SES families, but not in adolescents from low-SES families. Poor perceived health was also a stronger risk factor for low PA in males than in female adolescents. One possible explanation for these finding may be differences in conception of health. PA may be less strongly linked to health perceptions in low-SES adolescents and in females. In support of this notion, research has demonstrated that associations with self-perceived health were considerably different between native Dutch individuals and ethnic minorities [65], who are typically of lower SES [66]. These findings suggest that the meaning attached to the single-item question assessing self-perceived health may differ according to gender, ethnicity and SES.

Our findings also revealed that, while peer social support was a significant predictor of PA in all groups, it exerted the strongest effects on the PA of male adolescents from low-SES families, suggesting that peer social support may be particularly important for this subgroup. Research has found that children from high/middle SES schools report engaging in more family-based activities and organized sports than adolescents from low-SES schools [67]. In contrast, since costs are an important barrier for PA in adolescents from low-SES schools, this group reports engaging in more unstructured “free-play”, which is more influenced by peer social support [67]. Another study has shown that parental social support, an important predictor of adolescents’ PA [17], is lower in low-SES adolescents than in high-SES adolescents [68]. Given that other forms of social support for PA may be lacking in their home environment and that there are limited opportunities for family-based activities or organized sports, peer social support may be particularly important for promoting PA in adolescents from low-SES families.

Importantly, the three-way interaction with family SES and gender also suggested that, amongst adolescents from low-SES families, peer social support was also more important for males than females. This finding is in line with another study showing that peer social support was an important factor for PA in male adolescents, but not in females [29]. These results may reflect gender differences in motivation for PA. Given that fun of PA is a more prevalent motivation for PA in males than in females [22], and the fact that male adolescents are more likely to join sport clubs [69], it is plausible that peer social support has a stronger influence on the PA of male adolescents than females. The idea of “pick-up” games or neighbourhood activities may be more prevalent among males than females, and such activities may be more influenced by the peer social support. Certain environmental factors, such as neighbourhood safety, may also limit the opportunities for outside play with peers for females’ adolescents more than for males, particularly for those from low-SES families [70].

These findings emphasize the need of a multivariate approach to explore the factors associated with adolescent’s PA, as well as of how these factors may vary according to socio-demographic and -economic characteristics, since the interaction of these characteristics may give rise to unique experiences with different needs, barriers and facilitators for PA. One of this study’s strengths was that, rather than exclusively focusing on cognitive factors typically researched in the context of PA [50, 63, 71], this study assessed correlates of PA from various domains, namely socio-demographic and -economic characteristics, psychosocial, behavioural and environmental factors. Our results indicate that relevant factors from these different domains are not completely independent, but rather “intersect” or interact with each other. As suggested by intersectionality theory, gaining insight on how the influence of certain correlates on PA may vary, for instance, according to the adolescent’s gender or SES, can guide the tailoring of future interventions to address the specific needs of individuals. Another strength of this study included high participation rates and the systematic recruitment of a large sample of male and female adolescents from secondary schools, which ensured that our sample was representative of this target group and that our results can be generalized to other similar populations.

Several limitations of the current study should be considered when interpreting the results. It is important to note that this study adopted a cross-sectional study design, thus causality cannot be inferred for the associations observed. Longitudinal study designs are needed to further examine the link between sociodemographic characteristics, correlates of PA, and adolescent’s PA in order to gain more insight into the causality of these relationships. This was an explorative study in which the effect of multiple potential factors of PA and their interactions with gender and family SES were investigated. Hence, although the variables included were carefully selected by behavioural experts as potential predictors of adolescent’s PA prior to the start of the study, we recognize that multiple comparisons may have enhanced the chance of a type 1 error, which is a limitation of this study. Nonetheless, this explorative designed allowed us to explore whether correlates of PA in adolescents differed according to the intersection of gender and family SES, rather than examining these factors in isolation, which is an approach that has largely been overlooked. To guide the development of tailored PA interventions, further research is needed to replicate our findings and to continue investigating how the intersection of socio-demographic and -economic characteristics influences correlates of adolescent PA.

Another limitation inherent to research based on population surveys was the use of self-report measures. For instance, assessment of family SES relied on a single-item measure about experiencing difficulties with finances at home, which was a considerable limitation. Future studies, if possible, could utilize data on household income, or use validated questionnaires such as the Family Affluence Scale [72] to improve the validity of the findings regarding family SES. Additionally, although the survey incorporated a widely used and validated PA measure [35, 73], it is well established that self-report measures of PA are limited by recall difficulties, social desirability bias, and overestimation of PA levels, especially in adolescents [74, 75]. Future research is also needed to replicate our findings while implementing objective measurements of PA, such as accelerometers. Moreover, the single-item measure used assessed total levels of PA, which limited our ability to explore predictors of active-transport, recreational, and school-based PA separately. Future studies should collect data separately on these types of PA in order to explore the differential effects of various factors on certain types of PA. Nevertheless, a systematic review provided support for the use of short MVPA measures in large scale survey studies with youth [76]. Despite the limitations of the self-report measures used, the current study was not only able to identify several correlates of PA in adolescents, but also to gain insight on how these correlates of PA may vary according the intersection of gender and family SES.

## Conclusion

This study identified several factors that were associated with lower PA in adolescents. Moreover, we found that the association between certain factors and PA differed according to the intersection of gender and family SES. For example, peer social support was the strongest correlate of adolescents’ PA in our sample, but it had a stronger correlation with PA in male adolescents from low-SES families than in any other subgroups. Therefore, our findings suggest that PA interventions in adolescents may need to target different factors according to the adolescent’s gender and family SES in order to address the specific barriers and facilitators of PA in these subgroups. Future studies are needed to investigate how socio- demographic and -economic characteristics (e.g. gender, SES, migration background) can intersect and uniquely shape adolescent’s PA experiences, as well as to explore how the relevant factors for PA may vary for different subgroups according to these experiences.

## Supplementary Information

**Additional file 1.**

**Additional file 2.**

**Additional file 3.**

## Data Availability

The complete datasets supporting the conclusion of this article are not publicly available in an online repository, but can be made available upon request. Requests should be directed at the municipal health services (monitorgezondheid@ggdghor.nl).

## Abbreviations

PA: Physical Activity
SES: Socioeconomic Status
