# Supplementary material for: Exploring the intersectionality of family SES and gender with psychosocial, behavioural and environmental correlates of physical activity in Dutch adolescents: a cross-sectional study
Source: BMC Public Health. 2022 Aug 27;22:1623. doi: 10.1186/s12889-022-13910-6 (PMC9419391; doi:10.1186/s12889-022-13910-6)
Supplement: Supplementary file 2 — Additional file 2. [file 12889_2022_13910_MOESM2_ESM.docx]

**Supplementary Figure S2.** Predicted mean fixed scores of PA by self-perceived health stratified by family SES, with 95% CI. The model was adjusted for gender, age, migration background, educational level and municipality.
